# Jessie K., on Kissing

**Published:** 1889-05

**Authors:** 


### JESSIE K., ON KISSING.

Do, for goodness' sake, say something about the silly way that women have of kissing each other every time they get together. If twenty women were to meet in the street every last one of them would have to kiss the other nineteen, and there would be—let me see—380 kisses worse than thrown away. When you see one of these very violent miscellaneous kiss-everything-within-sight kind of women it is safe to set her down as a fraud, which she generally is.

